# Cerebral sinovenous thrombosis in children with nephrotic syndrome: systematic review and one new case

**DOI:** 10.3389/fped.2023.1207871

**Published:** 2023-08-24

**Authors:** Patrik Konopásek, Barbora Piteková, Vlasta Krejčová, Jakub Zieg

**Affiliations:** ^1^ Department of Pediatric Nephrology, 2nd Faculty of Medicine, University Hospital Motol, Charles University, Prague, Czechia; ^2^ Department of Pediatric Urology, Faculty of Medicine, Comenius University and National Institute of Children’s Diseases, Bratislava, Slovakia

**Keywords:** children, cerebral sinovenous thrombosis, pediatric, nephrotic syndrome, thromboembolism

## Abstract

**Aim:**

The aim of this review is to provide clinicians with characteristics of children with nephrotic syndrome and cerebral sinovenous thrombosis (CSVT).

**Methods:**

We have reviewed 37 articles of pediatric cases and provided 1 new case. PRISMA guidelines were followed.

**Results:**

Sixty-two patients were included in the review. CSVT was more common in males, usually occurred within 6 months of nephrotic syndrome onset and was found more often in outpatients. The superior sagittal sinus was the most common sinus affected. Non-contrast computed tomography was the most frequent radiologic study performed, with 30% of results negative for CSVT. Headache and vomiting were the most common symptoms while neurologic symptoms were less frequent. Anticoagulation treatment was strongly inconsistent throughout the literature. Thrombosis outcomes were favorable. The most common possible risk factors were corticosteroid treatment, proteinuria and hypoalbuminemia. Four children had a genetic predisposition diagnosed after thrombosis. No markers for anticoagulation prophylaxis seemed to be relevant for the majority of thrombosis occurring in outpatients.

**Conclusion:**

Prophylactic anticoagulation does not seem reasonable to prevent CSVT. Knowledge of nonspecific symptoms and of nephrotic syndrome being a state of hypercoagulation and early use of appropriate radiologic study seem to be of major importance.

## 1. Introduction

Nephrotic syndrome (NS) is defined as nephrotic-range proteinuria and hypoalbuminemia and is the most frequent glomerular disease in childhood (1, 2). NS is associated with a hypercoagulable state that is related to an imbalance between prothrombogenic and antithrombotic factors along with thrombocytosis and increased platelet activation. In addition, volume depletion, infection, severity of proteinuria, history of thromboembolism (TE), use of a central venous catheter (CVC) and corticosteroid (CS) therapy may predispose to a TE event, a well-recognized complication of NS (2–6). The incidence of TE is about 3% in the pediatric population which is less frequent than in adults. However, the incidence may be higher in some subgroups of children with NS like congenital or secondary NS (2, 3). Based on a recent systematic review, the pooled prevalence of TE is 3.6% in all forms of NS and up to 8.7% in congenital NS. Congenital NS forms together with steroid resistance were found to be potential risk factors for TE events. The most frequent histology present in more than half of children with NS and TE is focal segmental glomerulosclerosis (FSGS) (7). The most common vessels affected by TE are renal veins, veins of the lower extremities and pulmonary arteries (1, 8). Prior to CS treatment of TE, infections and acute kidney injury (AKI) were important causes of high mortality due to NS. Although CS improved the outcomes of NS in children significantly, TE remains a severe complication of the disease (2, 7, 9). Prophylactic anticoagulation treatment has been recommended in adult patients with membranous nephropathy based on serum albumin, arterial TE risk and bleeding risk stratifications (1). In children with NS, no proper approach to prevent TE has been developed so far as there is a lack of randomized trials to determine the efficacy and safety of prophylactic anticoagulation. However, there are a number of nonpharmacologic measurements that may decrease the risk of TE and these should be applied to patients with NS (3).

Cerebral sinovenous thrombosis (CSVT) in children is a rare condition with an incidence of 0.558–0.67 cases per 100,000 children per year (10, 11). The incidence in children with NS is unknown and varies among different studies (12–14). The presenting symptoms are mostly nonspecific. Thus, the diagnosis and therapy of CSVT may be delayed because the correct diagnosis requires a high index of suspicion in a child with associated risk factors (15–18). Various radiological studies may be performed to confirm the diagnosis of CSVT, with magnetic resonance venography (MRV) being the gold standard (17, 19). The aim of this review is to provide clinicians with clinical aspects and characteristics of children with NS and CSVT and to suggest proper management.

## 2. Methods

**Figure 1:**
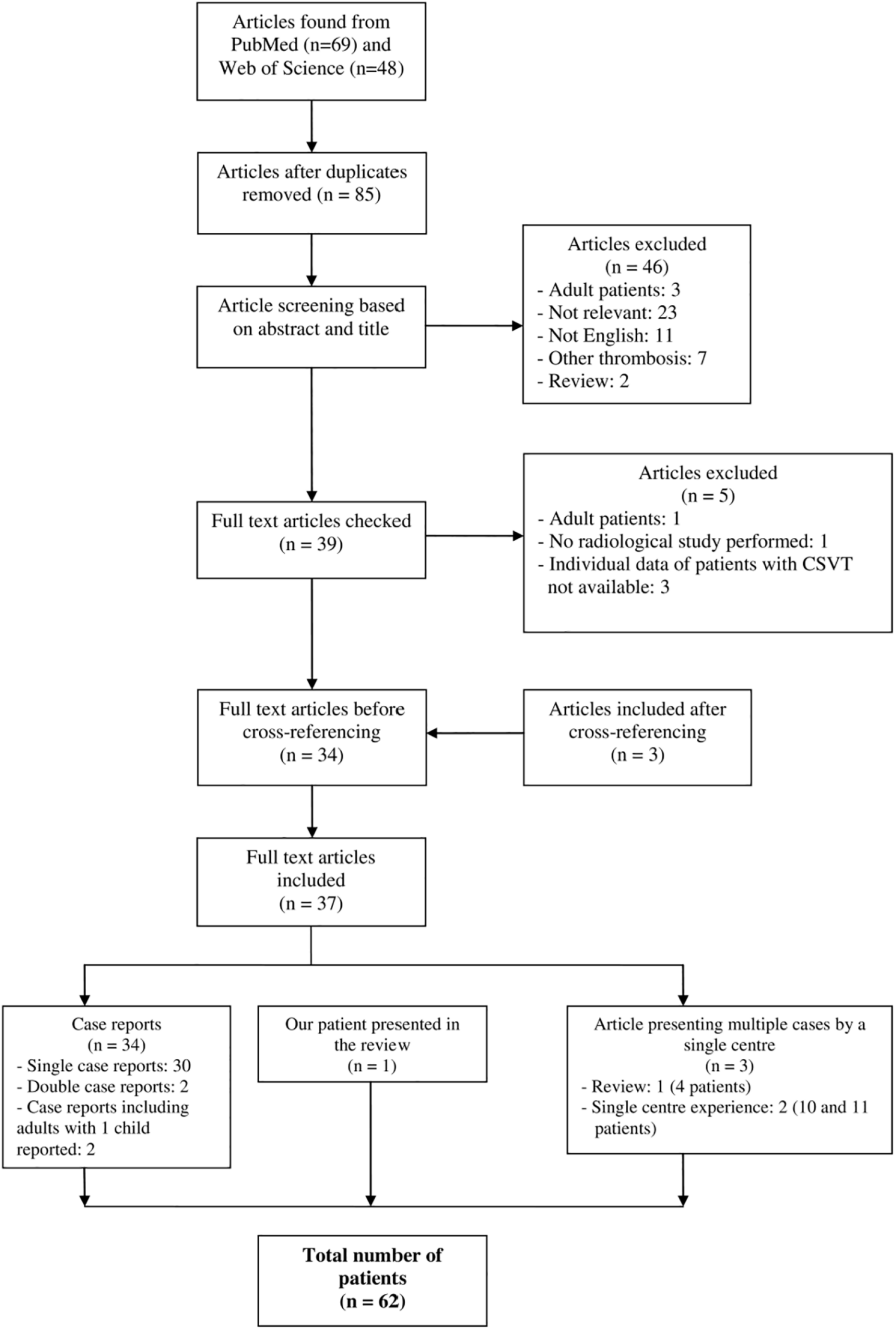
Flowchart.

PubMed/MEDLINE and Web of Science were searched and cross-referencing was done to identify publications relevant to NS and CSVT in the pediatric population. Medical subject headings (MeSH terms) used in the creation of the search strategy were “sinovenous thrombosis” or “sinus venous thrombosis” or “cerebral venous thrombosis” and “nephrotic syndrome” and “children”. Only articles relevant to the topic with sufficient data about each pediatric case (age 0–18 years) and with CSVT confirmed by any of the radiological methods were chosen. In case of review, only patients presented by the center were included. Thirty-seven articles met our criteria, consisting of 62 patients including our patient presented in the review (Figure 1) (8, 12, 16, 20–53). The first report was from 1980 (52). Each article was reviewed 3 times by 3 authors, creating a table of all relevant data (Supplementary Materials). The course of NS was defined as steroid-sensitive (SSNS) or steroid-resistant (SRNS). KDIGO definitions were used if the course of NS was not clearly stated by the authors (1). If any other information was not clearly stated by authors and not obvious from the text, it was described as unavailable (UA). Means + standard deviations and percentages were counted using available data. In the case of UA data, an absolute number of patients was presented in the format “number of patients with a value of the parameter/total number of patients with known value of the parameter”.

## 3. Results

### 3.1. Our patient

A 2-year-old boy was diagnosed with NS and went into remission after 3 weeks of prednisone treatment (60 mg/m^2^ daily). He relapsed on an alternate-day regimen and was given again a full daily dose of prednisone. After 4 weeks (2.2 months after NS onset) while he was still on daily prednisone without remission of NS, he developed acute vomiting. Accordingly, he was admitted to a local hospital and was given symptomatic treatment with intravenous isotonic solutions. After 3 days, he was transferred to our hospital for further evaluation of NS. At our department he did not vomit and he started to tolerate a small amount of oral liquids, but was denying solid food and remained irritable. He had nephrotic proteinuria and his serum albumin level was 24.5 g/L. On the third day in our hospital his symptoms deteriorated and he developed papilledema and slight nuchal rigidity. We performed acute MRV which showed massive CSVT affecting sigmoid, transverse, straight, superior and inferior sagittal sinuses and torcula. Low molecular weight heparin (LMWH) was started and his symptoms improved shortly after beginning anticoagulation therapy, with no neurological sequel. Because of the absence of NS remission after 6 weeks of daily prednisone treatment, a kidney biopsy was performed, showing minimal change disease (MCD). We started treatment with methylprednisolone pulses and a calcineurin inhibitor, reaching a remission in which he has since remained. The check-up MRV showed almost complete recanalization after 4.5 months and LMWH could be stopped as we found no risk factor for TE.

### 3.2. Basic characteristics

The mean age at CSVT onset was 6.17 ± 3.67 years. CSVT was more common in boys (80% vs. 20%; 49/61 vs. 12/61). Overall, 26% had primary or secondary SRNS (14/53) and 74% had SSNS (39/53); the course was not clear in 9 patients. Biopsy revealed MCD in 64% of cases (9/14), FSGS in 29% of cases (4/14) and immunoglobulin A nephropathy in 7% of cases (1/14). CSVT occurred during the first episode of NS in 34% of children (17/50) and in any subsequent relapse in 66% of them (33/50). CSVT was more common during the first 6 months from the onset of NS (67% of patients, 32/48). The latest CSVT occurred 96 months after NS onset. Thirty-one percent of patients (12/39) were inpatients and 69% (27/39) outpatients when they suffered CSVT. CSVT was diagnosed in less than a week in 81% of patients (35/43); in 1 case the diagnosis was made after 210 days. No case had a previous history of TE. Ninety-one percent (31/34) of children had unremarkable family histories, the grandfather of 1 patient had unknown thrombophilia and in 2 cases, TE occurred in 1 parent, each of whom was diagnosed with functional protein S (PS) deficiency.

On the day of CSVT diagnosis, 86% of patients (44/51) were on CS (3 of them on intravenous CS), of which 2 were also on cyclosporine A (CyA); 1/51 patients were on CyA without CS; 27% of patients (13/49) received diuretics. None of the reported patients had CVC inserted prior to CSVT.

### 3.3. Clinical course

Twenty-three percent of patients (14/62) had an infection or were vomiting during or prior to the CSVT episode (Table 1). Dehydration was described prior to CSVT in 7% of cases (4/61), 3 children were dehydrated on admission, probably due to vomiting or poor oral intake resulting from CSVT, and the timeline was not clear in 1 case of dehydration. Symptoms of CSVT were mostly nonspecific. The most common clinical signs were headache (69%) and vomiting (60%); in 15% of cases, vomiting and/or headache were the only symptoms. Papilledema was present only in 37% of children. Other symptoms suggesting central nervous affection were less common; the most frequent were oculomotor nerve palsy (26%), generalized seizure (24%), drowsiness/lethargy (19%) and irritability (11%). A complete list of symptoms is summarized in Table 1.

**Table 1:** Summary of clinical symptoms and treatments.

Clinical symptoms	% (n)
Headache	69 (43/62)
Vomiting	60 (37/62)
Papilledema	37 (23/62)
Oculomotor nerve palsy	26 (16/62)
Generalized seizure	24 (15/62)
Drowsiness/lethargy	19 (12/62)
Irritability	11 (7/62)
Positive meningeal signs	10 (6/62)
Focal seizure	8 (5/62)
Coma	8 (5/62)
Sensory loss	7 (4/62)
Diplopia	7 (4/62)
Nervus VII paresis	5 (3/62)
Hemiparesis	5 (3/62)
Status epilepticus	5 (3/62)
Dizziness	5 (3/62)
Altered consciousness after seizure	5 (3/62)
Ophthalmodynia	2 (1/62)
Hyperreflexia	2 (1/62)
Photophobia	2 (1/62)
Phonophobia	2 (1/62)
Stupor	2 (1/62)
Incoordination	2 (1/62)
Sequelae of CSVT	% (n)
Incontinence	2 (1/58)
Intermittent headache and vertigo	2 (1/58)
Low visual acuity	2 (1/58)
Mild right convergent strabismus	2 (1/58)
Strabismus with subsequent improvement after 6 months	2 (1/58)
Regression of self-care abilities, speech problems	2 (1/58)
Other sequelae	% (n)
Death due to massive PTE	2 (1/58)
Right arterial and vein gangrene after catheter insertion with subsequent leg amputation	2 (1/58)
Treatment	% (n)
VKA	69 (43/62)
UFH to VKA switch	36 (22/62)
LMWH to VKA switch	26 (16/62)
UFH to LMWH to VKA switch	5 (3/62)
tPA loc to tPA sys to UFH to LMWH to VKA switch	2 (1/62)
LMWH + UFH to VKA switch	2 (1/62)
LMWH	11 (7/62)
LMWH alone	8 (5/62)
UFH to LMWH switch	3 (2/62)
UFH	10 (6/62)
UFH or LMWH (not specified)	7 (4/62)
LMWH to tPA loc switch	2 (1/62)
Symptomatic treatment (no anticoagulation)	2 (1/62)
Additional treatment	% (n)
Urokinase	10 (6/62)
Dipyridamole	23 (14/62)
ASA	7 (4/62)
FFP	10 (6/62)
ATIII	2 (1/62)
Infection or vomiting prior to CSVT	% (n)
Fever	5 (3/62)
Temperature 37.5 °C	2 (1/62)
Gastroenteritis	5 (3/62)
Erosive gastritis	2 (1/62)
Vomiting	2 (1/62)
RTI + gastroenteritis	2 (1/62)
RTI	5 (3/62)
Upper RTI	2 (1/62)
Mastoiditis	2 (1/62)
Cough	2 (1/62)
Peritonitis	2 (1/62)

ASA, acetyl salicylic acid; ATIII, antithrombin III; FFP, fresh frozen plasma; LMWH, low molecular weight heparin; loc, local; PTE, pulmonary thromboembolism; RTI, respiratory tract infection; sys, systemic; tPA, tissue-type plasminogen activator; UFH, unfractionated heparin; VKA, vitamin K antagonist.

Ninety percent of patients (52/58) recovered completely after 1 to 60 days of anticoagulation treatment; 10% (6/58) suffered from sequelae of CSVT which were transient in 1 case and mild in most cases. In 1 child the right leg was amputated as a complication of right femoral artery catheterization. One patient died due to massive right pulmonary TE (PTE) with almost complete occlusion of the left pulmonary artery which occurred 20 days after CSVT onset on low dose heparin treatment (51).

### 3.4. Extent of thrombosis and parenchymal lesions

The superior sagittal sinus was the most frequently affected sinus (82%); the right side was slightly predominant (Table 2).

**Table 2:** Summary of radiological studies, sinus affections and laboratory results.

Radiological study	% (n)
ncCT	57 (35/62)
ncCT - CSVT dg	70 (21/30)
cCT	31 (19/62)
CTV	3 (2/62)
MRI and/or MRA	53 (33/62)
MRI and/or MRA – CSVT dg	86 (25/29)
MRV	52 (32/62)
Cerebral angiography	2 (1/62)
Laboratory study	% (n)
Albumin < 30 g/L	80 (36/45)
Albumin < 25 g/L	73 (33/45)
Albumin < 20 g/L	60 (27/45)
Proteinuria	90 (52/58)
Nephrotic proteinuria	74 (43/58)
Non-nephrotic proteinuria	14 (8/58)
Proteinuria of unknown severity	2 (1/58)
LA normal	100 (19/19)
aCA not present	100 (29/29)
DD > 250 µg/L	86 (12/14)
ATIII < 70%	26 (9/35)
Fibrinogen > 6 g/L	23 (7/30)
PC > 55%	100 (36/36)
PS < 55%	11 (4/36)
PLT > 450 × 10^9^/L	28 (11/39)
aAF not present	100 (6/6)
Urea > 6.7 µmol/L	32 (6/19)
Sinus affected	% (n)
SSS	82 (51/62)
TS	58 (36/62)
rTS	48 (30/62)
lTS	34 (21/62)
SS	34 (21/62)
rSS	29 (18/62)
lSS	21 (13/62)
SR	24 (15/62)
T	13 (8/62)
ISS	7 (4/62)
JIV	31 (19/62)

aAF, antiphospholipid antibodies; aCA, anticardiolipin antibodies; ATIII, antithrombin III; cCT, contrast-enhanced computed tomography; CSVT, cerebral sinovenous thrombosis; CTV, computed tomography venography; DD, D-dimer; dg, diagnosis; JIV, jugular and/or intracranial vein; LA, lupus antigen; lTS, left transverse sinus; MRI, magnetic resonance; MRA, magnetic resonance angiography; MRV, magnetic resonance venography; ncCT, non-contrast-enhanced computed tomography; PC, protein C; PLT, platelets; PS, protein S; rTS, right transverse sinus; SR, sinus rectus (straight sinus); SS, sigmoid sinus; ISS, inferior sagittal sinus; SSS, superior sagittal sinus; TS, transverse sinus.

There were 3 concomitant thromboses, PTE, a case of multiple thromboses (PTE + pulmonary artery occlusion + right auricula thrombosis), and PTE with thrombosis in the right femoral artery and vein as a complication of catheterization.

Twenty-one percent of patients (13/61) had some type of brain parenchymal lesions including bleeding, infarctions or nonspecific changes on radiological study.

### 3.5. Treatment of thrombosis

The treatment of CSVT was very inconsistent in the reviewed articles. Warfarin or other oral vitamin K antagonists (VKAs) were the most used anticoagulation drugs; the most commonly observed anticoagulation management was a short period of unfractionated heparin (UFH) or LMWH followed by long-term therapy with VKAs. Treatment was maintained for 3–12 months in the majority of patients (90%, 37/41). Interestingly, there was case describing spontaneous clinical improvement withqout anticoagulation treatment (Table 1).

### 3.6. Laboratory and radiological studies

In many cases more than 1 radiologic study was used. MRV was performed only in 52% of children (32/62). The most commonly used imaging method was non-contrast-enhanced CT, performed in 57% of cases (35/62), with 30% studies of negative for CSVT (Table 2).

Pathologic proteinuria and hypoalbuminemia were the most frequent laboratory abnormalities associated with CSVT (90% and 80%). Decreased antithrombin III (ATIII) < 70% and fibrinogen (Fg) > 6 g/L were less common (26% and 23%). Four children had reduced PS activity. Lupus anticoagulans, antiphospholipid and anticardiolipin antibodies were normal in all patients as was protein C (PC) activity. Genetic testing for thrombophilia showed factor V Leiden heterozygosity for G1691A mutation in 1 patient and functional PS deficiency in 3 children. Elevated D-dimer (DD) (>250 µg/L) was found in 86% of patients (12/14). Complete laboratory findings are summarized in the Supplementary Materials and Table 2.

## 4. Discussion

CSVT in children with NS was predominantly seen in boys, usually occurred within 6 months from NS onset and was not rare during the first episode. The children had mostly nonspecific symptoms (predominantly headache and vomiting). The patients were almost always on CS therapy and often outpatients during the CSVT episode. Proteinuria and hypoalbuminemia were described in the majority of the patients, while decreased ATIII and elevated fibrinogen were not frequent. Outcomes were favorable: the majority of children fully recovered without sequelae; 1 child passed away but the cause of death was not CSVT. The treatment of CSVT was very inconsistent and MRV as the gold standard in CSVT diagnosis was performed in only half of patients.

CSVT is a rare condition and the incidence in children with NS is unknown. Divecar et al. reported only 1 case from 700 patients with NS over 17 years (12). In a Bulgarian study, 9 from 447 children with NS had any TE over 22 years, with no CSVT reported (13). In a study by Sharaf et al., 4 children from 67 with NS suffered from CSVT (14). Symptoms of CSVT are often nonspecific such as headache, vomiting, fatigue, lethargy and irritability, with variable presentation. Especially in pediatric patients, signs may not be obvious and, in some cases, there may be no symptoms at all. Occasionally, we encounter specific neurologic symptoms, from motor impairment, visual alterations, cranial nerve palsies and focal or generalized seizures to coma (8, 15–19). Based on our data, the most common symptoms of CSVT in children with NS are vomiting and headache, symptoms which may be seen in many conditions (54, 55). In addition, headache may be difficult to assess in small children (55). A combination of nonspecific symptoms and rare incidence of CSVT leads to the misdiagnosis of this condition (53). The patient's condition can change rapidly within hours and can threaten their life; therefore, timely and correct diagnosis and appropriate treatment are essential (15, 53).

**Figure 2:**
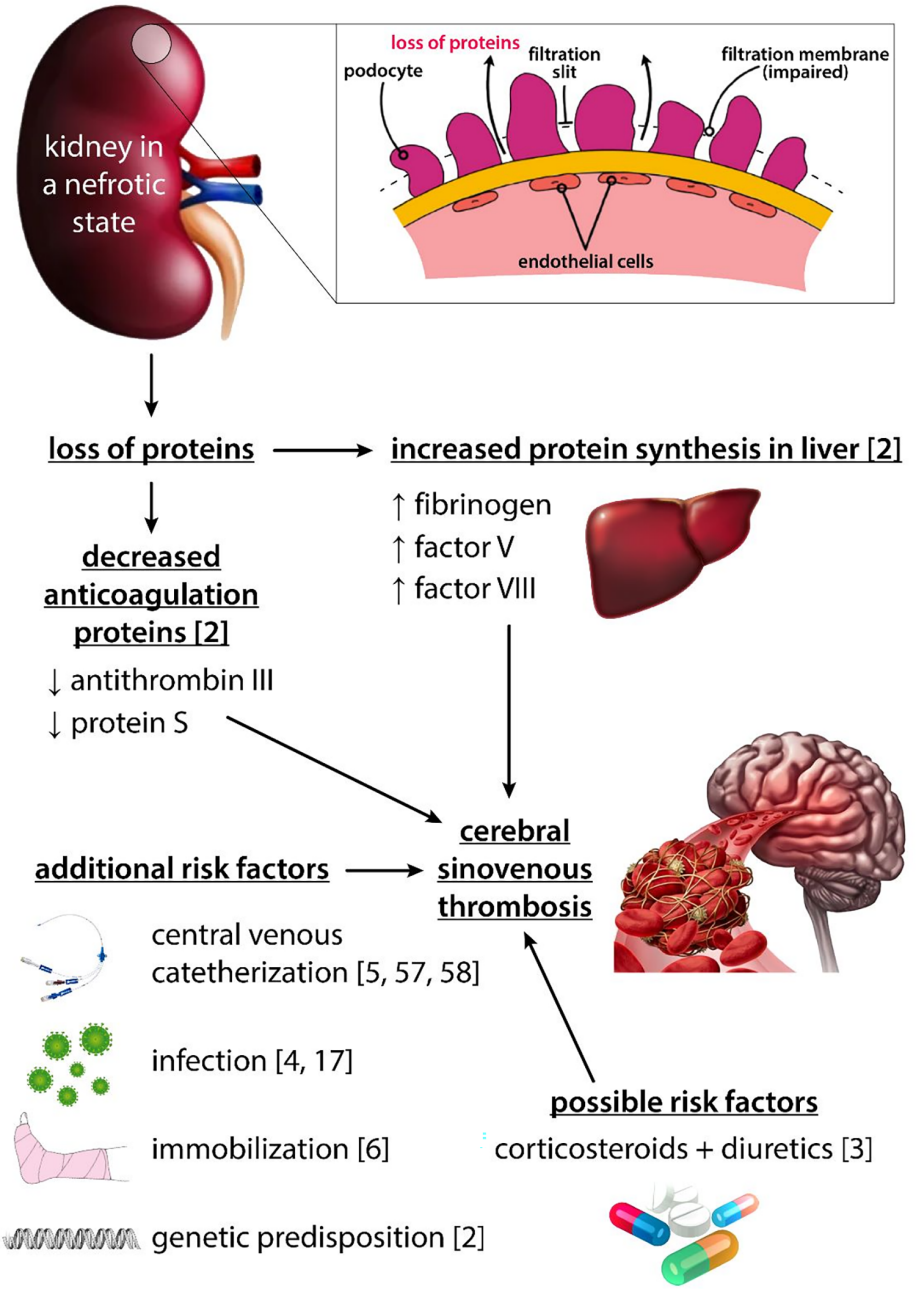
Pictorial representation of factors associated with TE in children with nephrotic syndrome.

There are many factors causing NS to be a hypercoagulation state (Figure 2). Due to glomerular damage, important antithrombotic proteins like ATIII and PS are lost in urine. On the other hand, increased liver protein synthesis leads to elevation of prothrombotic proteins with higher molecular weights (Fg, factor V, factor VIII) (2). CVC is one of the most important risk factors for TE and is associated with nearly 50% of deep venous thrombosis in childhood NS (5, 56, 57). One study described an infection to be the main cause of TE in children, with mastoiditis being the most frequent one (17). In one study on children with NS, the TE group had a significantly higher rate of infections compared with the non-TE group (4). Some patients may require immobilization which is a known risk factor for TE (6). Diuretics and CS have been linked to TE in children with NS, but it is not well established whether these agents are real risk factors as the majority of children with NS receive the treatment and none of these medications have been proven to be an important risk factor for TE in other diagnoses (3), Reactive thrombocytosis is common in NS and its role in the pathogenesis of TE is debatable (2). On the other hand, platelet aggregation and platelet adhesiveness have been proven to be increased in children with NS (2).

Despite NS being a hypercoagulation state, there is no clear recommendation on prophylactic treatment of TE (2). Identification of strong predictors for TE would only help to treat children with a high risk of thrombosis. Previous history of TE, age >12 years and severe proteinuria are strongly associated with TE; other factors are membranous nephropathy, systemic lupus erythematosus and secondary glomerulopathies (5). Current KDIGO guidelines recommend considering prophylaxis in the case of albumin level <25 g/L and additional risk factors, but previous studies did not find hypoalbuminemia to be associated with TE in childhood NS (1, 5). Some authors recommend prophylaxis in the case of laboratory signs of hypercoagulability (albumin <20 g/L, Fg > 6 g/L, ATIII < 70%) (9). In the present review, no patient had CVC inserted and infections were not common. Only 3% of children were >12 years, 4 had genetic predisposition and no child had a history of TE. Hypoalbuminemia was a very frequent potential risk factor, but 69% of children suffered CSVT as outpatients. Thus, the albumin level was usually UA for the decision about prophylactic treatment. In the majority of cases, CSVT occurred within 6 months of NS onset, which has already been reported (1). Most of the children were on CS and had pathologic proteinuria, which may be assessed at home by a dip stick, and CSVT was more prevalent in boys. Based on these data, the only indicators for CSVT prophylaxis would be time from NS onset (6 months), presence of proteinuria, CS treatment and male sex. That would cause extreme overuse of prophylaxis just to prevent very few cases (in our review 62 cases in about 40 years in English written literature), and the prophylaxis would cover only some CSVT cases. Another aspect is that in the present review, outcomes of CSVT were favorable. Based on that, more focus should be put on early recognition of CSVT by clinicians, who should be aware of the hypercoagulation state in NS and nonspecific symptoms of CSVT and use timely proper diagnostic tools. A normal DD make the presence of CSVT very unlikely. However, if the patient is considered clinically likely to have CSVT, the diagnosis should still be suspected regardless of DD value (58, 59). In the present review, 2 out of 14 patients had DD < 250 u/L.

Neurological imaging methods are the basis for diagnosing CSVT. Brain CT is usually the first examination due to its availability and quick execution. Also, the clinician usually does not consider CSVT as the cause of the patient's difficulties in the first instance (3, 10, 19). Brain CT may show cerebral infarctions–initially, hyperdensity caused by a thrombus called the “cord sign”, followed by hypodensity caused by a defect of blood flow called the “empty delta sign” (60). However, up to 40% of patients have a cerebral CT with normal finding and therefore the absence of pathology on CT does not rule out CSVT reliably (61). These results are similar to those of the current review. The gold standard to establish the diagnosis is MRV, showing us exact cerebral venous and sinus flow in combination with MR of the brain. Therefore, in the case of clinical suspicion of CSVT, these methods represent the first modality to be used (60). If it is not possible to perform MRV immediately, CT venography (CTV) could be performed with caution of potential nephrotoxicity (18).

UFH, LMWH and VKAs are used as a treatment for TE in children (57). UFH with a short half-life and quick onset of action is often used in critical care. Also, it seems that heparin-induced thrombocytopenia is less common in children. LMWH is usually administered twice daily subcutaneously, maintaining anti-Xa levels between 0.50 and 1.0 U/ml at 2–6 h. It is an anticoagulant of choice in pediatrics even if the predictability of the treatment effect is less than in adults (57). VKAs are medications with a need for close monitoring, that may be difficult in children and therefore these are often avoided in infants for different reasons (57). In the current review, the treatment of CSVT was very inconsistent, with VKAs being the most common drug used. One reason may be the long period of time between the first and last paper included in our review (40 years).

**Figure 3:**
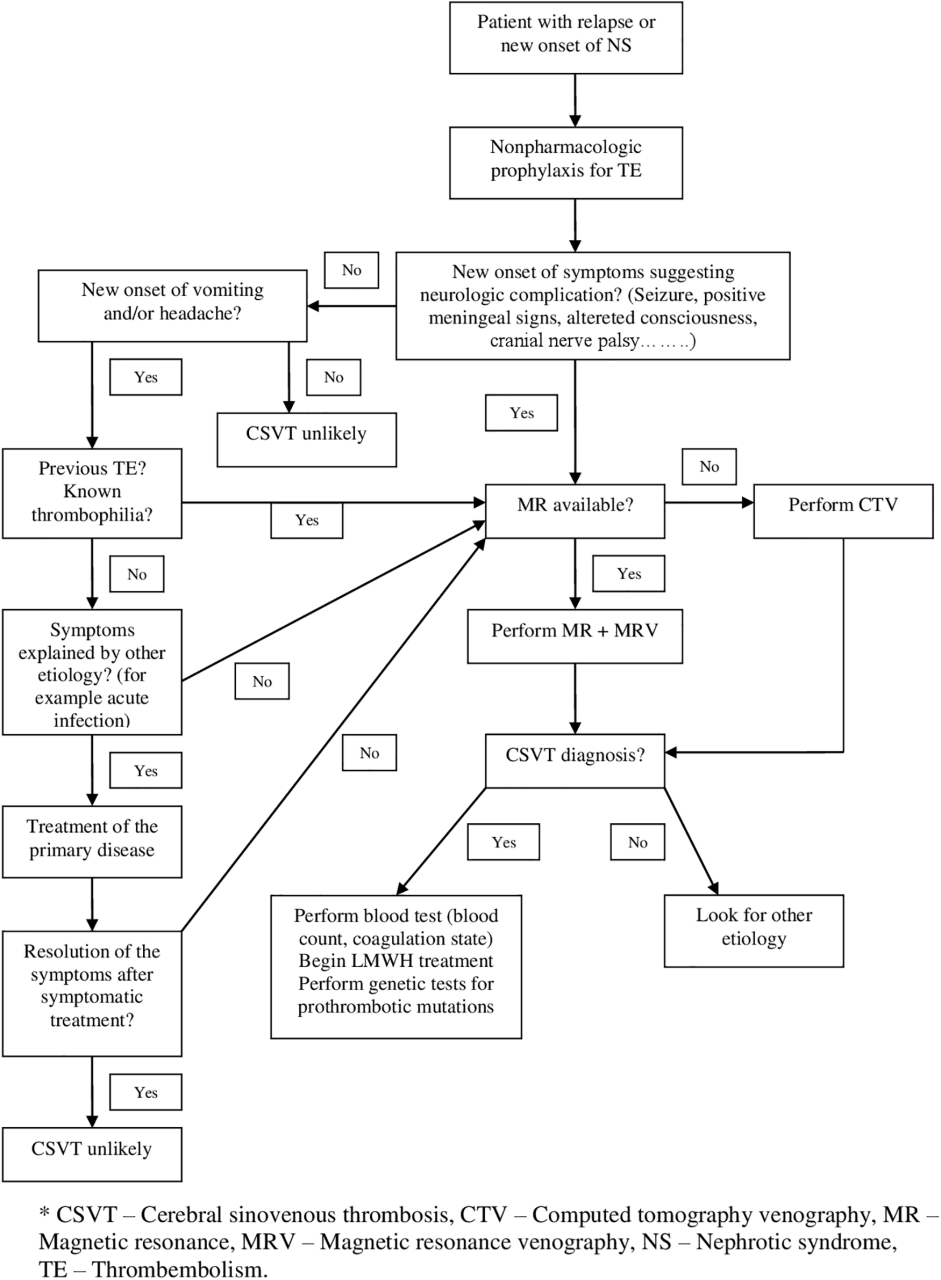
Diagnostic and therapeutic approach for suspected CSVT in children with NS.

Based on the combination of up-to-date information and results from our systematic review we prepared the diagnostic-therapeutic approach for clinicians An early recognition of nonspecific signs of CSVT with a subsequent appropriate choice of an imaging method to confirm our clinical suspicion should be the basis of diagnosis of CSVT in children with NS. The next steps in management of confirmed cases of CSVT in children are the realization of blood tests and starting LMWH treatment as soon as possible (Figure 3).

In conclusion, CSVT is a rare complication of NS. It seems to be more reasonable to focus on early recognition (be aware of nonspecific signs of CSVT), correct diagnosis and treatment of the condition than on prophylactic treatment. It is also important to select the most appropriate imaging for the correct CSVT diagnosis to avoid unnecessary and nonspecific examinations. Outcomes of CSVT in children with NS seem to be favorable; nevertheless, itis necessary to promptly diagnose and quickly treat patients with CSVT to prevent further progression of the disease.
